# Bipolar Chronobiology in Men and Mice: A Narrative Review

**DOI:** 10.3390/brainsci13050738

**Published:** 2023-04-29

**Authors:** Nadja Freund, Ida Haussleiter

**Affiliations:** 1 Division of Experimental and Molecular Psychiatry, Department of Psychiatry, Psychotherapy and Preventive Medicine, LWL University Hospital, Ruhr-University, 44791 Bochum, Germany; nadja.freund@ruhr-uni-bochum.de; 2 Department of Psychiatry, Psychotherapy and Preventive Medicine, LWL University Hospital, Ruhr-University, 44791 Bochum, Germany

**Keywords:** circadian, translational, sleep

## Abstract

In patients with bipolar disorder, we do not only see a cycling of mood episodes, but also a shift in circadian rhythm. In the present overview, the circadian rhythm, the “internal clock”, and their disruptions are briefly described. In addition, influences on circadian rhythms such as sleep, genetics, and environment are discussed. This description is conducted with a translational focus covering human patients as well as animal models. Concluding the current knowledge on chronobiology and bipolar disorder, implications for specificity and the course of bipolar disorder and treatment options are given at the end of this article. Taken together, circadian rhythm disruption and bipolar disorder are strongly correlated; the exact causation, however, is still unclear.

## 1. Bipolar Disorder

Bipolar disorder (BD) is characterized by depressed, manic, or hypomanic episodes with specific changes in physical activity, circadian rhythm, and sleep. Disruption of sleep and circadian rhythm disruption (CRD) are key features of BD and generate highly prevalent symptoms [1]. These disturbances encompass a range from symptoms to prodromal signs to treatment targets [2]. Rodent models for BD therefore also often show CRD or are even generated by specifically manipulating the circadian rhythm [3,4].

## 2. Circadian Rhythm

Circadian rhythms have initially been described as parameters of the organism’s time structure acquired through evolution on earth. In 2017 Hall, Rosbash, and Young received the Nobel Prize in Physiology and Medicine for their discoveries of how plants, animals, and humans adapt their biological rhythm so that it is synchronized with the earth’s revolutions. They discovered period and timeless proteins as well as underlying clock genes such as doubletime. Today, more than 20 clock genes have been discovered. These genes generate cellular circadian rhythms via intracellular transcriptional feedback loops. Within the animal kingdom, some very specific regulations of circadian rhythms have evolved. A few animals show phases of activity around the clock [5], and arctic reindeer, for e.g., do not have a circadian rhythm in winter and summer [6]. Bumble bee queens lose their circadian rhythms with motherhood [7], and the annelid polychaete worm Platynereis dumerilii adapts its circadian rhythm to the moon phases [8]. However, a lot of species, including man and mice, constitute circadian systems which exhibit rhythmic changes with about one cycle a day [9]. Circadian rhythms are highly conserved among mammals [1]. However, very interestingly, distinct sex differences within circadian systems have been reported and might be especially relevant for disease treatment (for review see: [10]).

## 3. Circadian Clocks

Mood regulation and circadian clocks are connected via biological pathways. The biological clock regulates our genes and, subsequently, the circadian rhythm adapts our physiology to the different phases of the day. The circadian clock affects the expression of clock-controlled genes that are rhythmically expressed and regulate crucial physiological pathways within the intrinsic circadian period of 24 h (stress pathways, serotonin, dopamine, mitochondria, inflammation, melatonin, and calcium channels) [1]. Circadian clocks require external time signals, with light perception being the most salient one [11]. The pathways linking the retina and the pineal gland are channeled through the suprachiasmatic nucleus (SCN) and the hypothalamic paraventricular nucleus, with glutamate being the major neurotransmitter in the retinal afferents. Photic signals from environmental light directly entrain the SCN with a specific subset of melanopsin-expressing, intrinsically photosensitive retinal ganglion cells that signal directly to the hypothalamus via the retinohypothalamic tract [12]. Subsequently, the SCN orchestrates the alignment of all molecular peripheral clocks via the rhythmic downstream expression of various proteins [13]. Thus, the mammalian circadian clock within the SCN also regulates the pineal gland metabolism and melatonin secretion. As early as in the 1970s, lesion studies in rats revealed the important role of the SCN in controlling the circadian rhythm [14,15]. Later on, electrophysiological studies confirmed that the SCN retains rhythmic electrical activity, even in the absence of light cues and even under in vitro conditions [16]. Within the SCN, individual cells generate a circadian rhythm via an interplay of multiple transcriptional and translational negative feedback loops coordinating rhythms across the entire body. The loss of rhythm or coordination among brain regions would generate critical changes in behavior, hormone levels, sleep, body temperature, affect, and metabolism [1]. CRD may emerge from any point in this system.

Melatonin secretion as a key mechanism coordinates circadian rhythm across the brain and peripheral systems. Melatonin synthesis and alterations thereof seem to be associated with BD [17,18]. Time of production onset as well as melatonin levels in BD patients differed between depressive and healthy subjects. These findings occurred not only phase-dependent, but also in euthymic states [19]. Recovered BD patients showed lower levels of melatonin, whereas manic BD patients displayed elevated daytime levels and earlier onset of production [20,21]. One study found that BD patients show lower levels of 6-sulfatoxymelatonin in first-morning urine samples compared to controls, a difference not found in MD patients [22] and suggesting a phase-shift of melatonin production.

Melatonin suppression resulting from hypersensitivity to nocturnal light has previously been considered a BD trait marker [20].

Inflammation and stress are bidirectionally connected to circadian rhythms and may be involved in BD pathophysiology [23]. CRD increases inflammatory cytokines [24] and vice versa. Inflammation induces phase delays in the SCN via altered gene expression [25] and the circadian clock interfaces with stress pathways [26].

## 4. Circadian Rhythm Disruption and Phase Shifting

Disruption of sleep and CRD are key features of BD and generate highly prevalent symptoms. CRD has been observed in several high-risk, prodromal, and syndromic psychiatric states and seems not to be specific to BD.

However, distinct circadian abnormalities lead to a measurable behavior pre-onset, cause specific symptoms and episodes, and might even identify BD subgroups. The delay or advance of circadian phases generates the polarity of BD phases [1]. Phase-shifting of circadian rhythms can be accomplished by a change in the institutional routine of human subjects or by the reversal of the lighting regimen in the experimental animal room [9]. Phase shifts have been associated with mood episodes in BD, where the phase delay increases along the clinical symptom continuum of intense emotional states from depression to mixed states to mania. Differences in the range of phase shifts might explain differences across the affective spectrum; BD I patients show the greatest phase shifts, BD II are intermediate, and unipolar depressed patients have the smallest phase shifts, suggesting both trait and state-like aspects of circadian phase shifting in BD. Phase advance, as well as delay, have been shown to resolve with effective treatment [27].

In models of nocturnal rats, an extended active period (5 h of light) decreases anxiety and immobility in the forced swim test, while shortening the active period (19 h of light exposure) results in more anxiety behavior in the elevated plus maze and more depressive-like behavior measured by increased immobile time in the forced swim test [28]. The long light period is accompanied by a switch from dopamine to somatostatin expression in hypothalamic neurons, while the contrary effect is observed after a short light period. In line with this involvement of the dopaminergic systems is the finding that mice with lower dopamine transporter expression show more pronounced behavioral effects [29] after phase-shifting. Even dim light stimulation during the dark phase can alter circadian rhythms and the expression of genes involved in the circadian rhythm and induce depressive-like behavior in mice [30]. In conclusion, phase advances are concomitant with manic BD phases, whereas depression involves a phase delay. It remains uncertain whether these changes represent a trait or state in BD.

## 5. Sleep

Sleep is a recurring biological process and a reversible state of decreased consciousness, perceptual disengagement, physical quiescence, recumbent posture, and eye closure [31]. Sleep serves the human body for restoration and cognitive functioning. Sleep disturbances, such as insufficient duration or quality, and irregular sleeping patterns, constitute frequent and serious symptoms of affective disorders [32]. BD patients have a significantly worse perception of their sleep, even in euthymic phases [33]. Mood disorders and biological rhythms might have overlapping underlying genetics, but it remains unclear whether the observed phase shifts during affective episodes are the cause or consequence of the given mood episode. [34]. CRD might be a common underlying factor that bridges mental health disorders and mediates similar clinical phenotypes [35]. The sleep–wake cycle is the most conspicuous process following a circadian pattern and is regulated by homeostatic and circadian principles, with core body temperature and melatonin levels being the most reliable indices. Sleep homeostasis is represented as a sleep pressure that increases during wakefulness and dissipates during sleep. If wakefulness is extended beyond the typical range, then a sleep debt is created [31]. Sleep phase and amplitude are strongly influenced by circadian function, whereas sleep latency, staging, and overall quality are less related. Sleep disturbance occurs prior to both depressive and manic phases, with actigraphy data demonstrating differences in sleep latency, sleep duration, wake-after-sleep onset, and sleep efficiency [36]. Very interestingly, total sleep deprivation (staying awake for about 36 h) in bipolar depression seemed beneficial as an add-on to medication in a recent meta-analysis [37].

By screening randomly mutagenized mice, two very specific sleep-related phenotypes were found. One mutant called Sleepy displays an increased need for sleep but with an unaffected circadian system. The mutant Dreamless shows reduced REM (rapid eye movement) sleep and a reduced circadian period length under complete darkness [38]. Unfortunately, these mutants seem not to have been characterized in terms of mood-related behaviors.

Sleep deprivation, however, has been well-studied in rodent models. In very early studies, rats had to sit on flowerpots surrounded by water and as soon as they would enter REM sleep, they would fall and wake up. Contrary to the hypothesis, these animals had no impairments in avoidance learning but showed increased activity and increased sensitivity to environmental stimuli according to the authors’ interpretation [39]. In the meantime, this animal model has further been characterized, and an increase in novelty seeking [40], reduced anxiety [41], and increased aggressiveness [42] and sexual behavior [43] indicate an overall mania-like behavior following sleep deprivation. Underlying neurobiological mechanisms have been associated with mitochondrial dysfunction [44], inflammation [45] in rats, and synaptic plasticity in mice [46]. In contrast, chronic sleep deprivation induces anxiety behavior in rats [47] and mice [48].

Taken together, acute sleep deprivation is highly associated with mania-like behavior; however, it has to be considered that the deprivation protocol always includes the induction of stress [49], and stress is considered a risk factor for BD.

## 6. Risk Factors and Genetics

Studies of circadian rhythms and vulnerability to BD in variously defined high-risk groups (familial, prodromal, temperamental) found a more variable sleep duration and altered sleep latency and quality in people with familial risk [50,51,52,53], delayed and fragmented activity, and a more variable irregular social rhythm with lower relative amplitude of activity patterns in people with hypomanic symptoms [54,55].

Daytime sleepiness correlated positively with affective disorders (major depressive disorder (MDD) as well as BD) in a recent gene-based analysis. MDD and BD-I differed the most regarding circadian rhythm, with BD-II taking up an intermediate position. It differed significantly from BD-I in relative amplitude and daytime sleepiness and from MDD in moderate activity. BD-I was positively associated with overall physical activity and negatively associated with sedentary behavior, whereas MDD and BD-II were negatively associated with the relative amplitude of the circadian rhythm [31]. Regarding the relative amplitude, BD-I and BD-II differed considerably: there was a negative genetic correlation of relative amplitude with both MDD and BD-II, but not with BD-I [56,57]. Self-report measures of sleep duration had a significant genetic correlation with BD but not MDD [58], and there was no significant genetic correlation with either MDD or BD when considering objectively measured sleep duration. All analyzed affective disorders significantly positively correlated with daytime sleepiness [34]. In concordance with the clinical picture, all affective disorders resembled each other regarding daytime sleepiness. Sleep duration, relative amplitude, and daytime sleepiness were similar to each other in MDD and BD-II, whereas BD-I and BD-II showed similar parameter values of (increased) physical activity. BD exists on a spectrum that may include temperamental features, and hyperthymic temperament has been proposed as a risk factor for BD. Evening chronotype is associated with BD risk phenotypes in general and with all affective temperaments as well, except hyperthymic temperament (association with morning chronotype) [59,60].

In animal models, recent advances in genetic manipulation have allowed for the thorough analysis of genes and pathways involved in circadian rhythm and behavior associated with BD-like behavior. The best characterized model is the ClockΔ19 mutant mouse. Here, an antimorph mutation on exon 19 of the Clock gene [61] causes an extension of the circadian period and loss of rhythmicity [62]. In addition, behavior that can be interpreted as mania-like behavior is displayed by ClockΔ19 mutant mice. Compared to wild-type animals, they show anxiety- and depression-like behavior, increased impulsivity and reward-seeking, locomotor hyperactivity, as well as impaired decision-making and sensorimotor gating [63,64,65,66,67]. Very interestingly, these behaviors are only present when tested during the day (light/inactive phase) but not during the active, dark phase [68]. Neurobiological alterations, including increased dopaminergic activity and glutamate levels, further validate the ClockΔ19 mutant mouse as a model for BD [69]. Even a knockdown of Clock gene expression specifically in the ventral tegmental area using a viral approach with RNA interference can disturb the circadian rhythm in these animals, indicated by less robust activity during the dark phase and enhanced activity in the resting phase. In addition, animals show reduced anxiety behavior. However, in contrast to ClockΔ19 mutant mice, they also display increased depressive-like behavior [70]. The second core component in the primary feedback loop to control circadian rhythms is Mop3 (also later named BMAL1) [71]. Mice with a knockout of this gene rely on light stimulation to keep a circadian rhythm. They show reduced activity [72], impairments in short- and long-term memory [73], impaired locomotor activity, reduced novel object and social recognition, and reduced reaction to cocaine (sensitization, conditioning, self-administration) [74].

Target genes of CLOCK and BMAL1 are Period and Cryptochrome. Inactivation of Period1 in mice induces a reduction of the active period [75] and depressive-like behavior [76]. Depressive-like behavior has also been shown in mice after knockout of Period2 [77], crypotochrome1 [78], and both crypotochrome1 and crypotochrome2 [79].

## 7. Environment

Our well-being is affected when there is a temporary mismatch between the external environment and internal biological clock. External zeitgebers (such as variation of body temperature, timing of meals, physical exercise, and social interaction) constitute key components in the bipolar pathogenesis, pathophysiology, and phenotype [1]. So does the hypothalamic–pituitary–adrenal axis, which follows a clear circadian variation and differs between those with and without BD [80]. Early environmental light exposure was associated with age of onset, suicidality, and manic and depressive features over the course of BD [81,82]. The human brain keeps track of the seasonal changes in the photoperiod using the SCN as a seasonal clock, which measures the length of daylight and adapts circadian rhythms accordingly. Seasonal changes affect all patients with mood disorders, and seasonal affective symptoms occur more frequently in BD compared to MDD or community controls. Previous studies suggested that subjects with BD II were the most likely to show seasonal mood variation and disability compared to MDD and BD I [83]. Genome-wide association studies (GWAS) observed associations between seasonal BD and clock gene variants, especially with NPAS2, CSNK1E, and RORA [84,85]. There is no conclusive evidence of whether light exposure or individual differences in light processing play causal roles in BD. Ambient light may have important effects on mood including the onset of manic symptoms and current depressive symptoms [86]. Shift work induces phase changes in susceptible people, and CRD in BD patients working at night caused mood disturbances and increased risk of relapse [87]. Occupational challenges such as shift work or school schedules can produce chronic mismatch between the circadian clock and the environment [2]. According to the social zeitgeber theory, life stress disrupts external social zeitgebers such as variable sleep, wake time, or mealtimes, and thus affects the internal biological zeitgebers. This can then disrupt the circadian rhythm, leading to mood episodes [88].

In rodents (rats and mice), stress has been shown to influence the activity rhythm as well as molecular markers of the circadian rhythm [89].

## 8. Bipolar Specificity and Course of Illness

CRD does not have a unique genetic association with BD but is equally important in the etiology of MDD and schizophrenia. Low-amplitude activity rhythms and altered sleep duration as risk factors have been observed in both affective and psychotic disorders [1]. So far, there are not distinct biomarker profiles to enable a nosological attribution. Actigraphy studies observe lower activity levels in individuals with MD [90] and BD [91]. When comparing the different mood states (depressed, manic, hypomanic) within BD, significantly higher levels of activity during manic and hypomanic phases are observed than in depressed phases [92]. GWAS found positive associations of bipolarity with physical activity, but none with daytime sleepiness [93,94].

Even though CRD may trigger a first affective episode, subthreshold manic symptoms proved the strongest predictor of transition to BD. Irregular social rhythms predicted conversion to BD, whereas sleep–wake inversion, anergy, and hypersomnia did not [95]. In conformity with these results, stressful life events, which cause disruptions in individuals’ social routines, have been shown to precede BD phase onset (manic phase in BD I and depressive phases in subthreshold BD and BD II) [96].

Findings are controversial as to whether circadian disruption in MDD might predict later transition to BD: one study found diurnal mood variation as baseline symptoms of MDD to be associated with the later transition to BD [97], whereas another study named depressive symptoms such as anergy and hypersomnia to be specifically associated with transition [92]. Every third BD patient suffers from a comorbid loss of synchronicity between the sleep–wake cycle and the day–night cycle, and a delayed sleep–wake phase is particularly common in BD. The shift in the circadian phase toward eveningness has been associated with earlier age of onset, increased suicidal behavior, higher relapse rates, and more drug-induced switching I BD, thus indicating a more severe course of illness [98]. Persisting eveningness in BD patients was observed longitudinally (even in remission) and associated with worsened quality of life, an impairment in interpersonal relationships, and a poorer prognosis [99].

## 9. Treatment

Treatment guidelines recommend lithium as the gold standard treatment for BD with anticonvulsant, antipsychotic, or antidepressant medications also used adjunctively depending on the episodic phase of the disorder [100]. Nevertheless, lithium is not consistently effective, and only approximately every third patient shows a favorable responsiveness. Prediction biomarkers such as polygenic scores should be identified, since they might be able to predict a more severe course of BD and could also establish individual treatment options [101]. Chronotype at baseline was associated with lithium response in BP I patients, and stronger morningness was favorable in the long run. A positive lithium response is predictable through GWAS, in which non-responders showed a longer circadian period and a lower amplitude of circadian rhythm [102]. Recently, a respective polygenic score for lithium treatment response (Li+PGS) in patients with BD has been developed [101]. Lithium seems to exert period shortening/shifting effects and trait-like differences in cellular rhythms that might influence its effectiveness and the patient’s response [102]. Overall, the therapeutic manipulation of either the phase or period seems promising: a phase advance might evoke antidepressive effects, whereas a phase delay would have an antimanic effect. Singular antidepressant interventions in BD (sleep phase advance, sleep deprivation, tricyclic antidepressants) as well as combined chronotherapeutic approaches (phase advance with lithium, phototherapy, or sleep deprivation) have shown phase advances and synergistic outcomes [103]. Fast-acting treatments such as short-term sleep deprivation, bright light therapy, and ketamine address the overlapping pathways which regulate mood and circadian circuits equally [104]. Interestingly, lithium is able to normalize the behavioral phenotype of rats after sleep deprivation [105], of ClockΔ19 mutant mice [106], and cryptochrome and period mutant mice [78].

## 10. Conclusions

Taken together, multiple studies in human patients as well as animal models confirm a strong relationship between CRD and BD. In animal studies, the manipulation of sleep and circadian rhythm as well as pathways involved in circadian rhythm induce behavior that is associated with bipolar disorder. Nevertheless, the exact mechanism and causation in the human condition are still unclear. Future clinical as well as preclinical studies to investigate this relationship are highly needed. Specifically, the development of treatment options addressing circadian rhythms seems very promising.

## Data Availability

Data sharing not applicable.
